# Cricoid malformation and endolaryngeal submucosal drilling – a possible technique

**Published:** 2017

**Authors:** A Zamfir-Chiru-Anton, AE Stanciu, DC Gheorghe

**Affiliations:** * ENT Department, “G. Alexandrescu” Children Hospital Bucharest; ** Research Department, Oncology Institute of Bucharest; *** ENT Department, “M.S. Curie” Children Hospital, Bucharest; “Carol Davila” University of Medicine and Pharmacy, Bucharest

**Keywords:** laryngeal malformation, cricoid cartilage, single stage laryngoplasty

## Abstract

Cricoid malformations vary according to their severity and anatomic features. Some of them get a delayed diagnosis in spite of the complex medical care.

**Objective:** To present a case with a laryngeal malformation and our surgery technique.

**Material, method:** A case presentation of a cannulated child, who previously had a heart surgery, with difficult decannulation. Results: The patient was considered to have an acquired subglottic stenosis due to his medical record. A cricoid cartilage malformation was elicited intraoperatively. A submucosal drilling of the redundant cartilage, with a preservation of the covering laryngeal mucosa was performed. Decannulation was possible at 13 days after surgery.

**Conclusion:** Instead of having extensive resection surgery we decided to perform an endolaryngeal submucosal drilling, after laryngofissure, with quick and long lasting success. Laryngeal surgery should always be tailored to the patient needs and the surgeon’s preferences.

## Introduction

Malformations of the larynx can manifest either at birth or later in infancy. The dimension of the laryngeal inlet decides if the respiratory distress requires an early or late intervention for the stabilization of the airway. Multiple possible lesions can be found and account for different grades of respiratory obstruction and clinical symptoms.

Cricoid cartilage anomalies are uncommon and not very well described in the literature, from a histologic point of view. Most of them are associated with congenital subglottic stenosis. This condition represents 10-15% of laryngeal malformations [**1**]. It has been associated with different anatomic lesions:

- elliptical cricoid cartilage, most frequently described [**2**-**4**];

- cricoid cleft, either anteriorly or posteriorly located [**5**,**6**];

- trapped first tracheal ring [**4**].

These cases can present with stridor at birth if severe enough or manifest later in childhood, after an upper respiratory tract infection or a medical intervention of the airway. Sometimes, the differential diagnosis can prove challenging. Suspicion is the main reason for the discovery of the disease and of fully understanding the nature of the patient’s symptoms.

We presented a case that demonstrated the silent and hidden nature of the cricoid lesions. They went unrecognized for a long period, until a specialized ENT department was able to identify the problem and decide to surgically approach the problem.

## Case presentation

A 4-year-old boy presented for admission to the ENT department, with a tracheal stoma installed after a congenital heart surgery into another hospital, 1 year before. The medical history did not reveal any other abnormalities but previous repeated attempts for decannulation, without success.

**Fig. 1,2:**
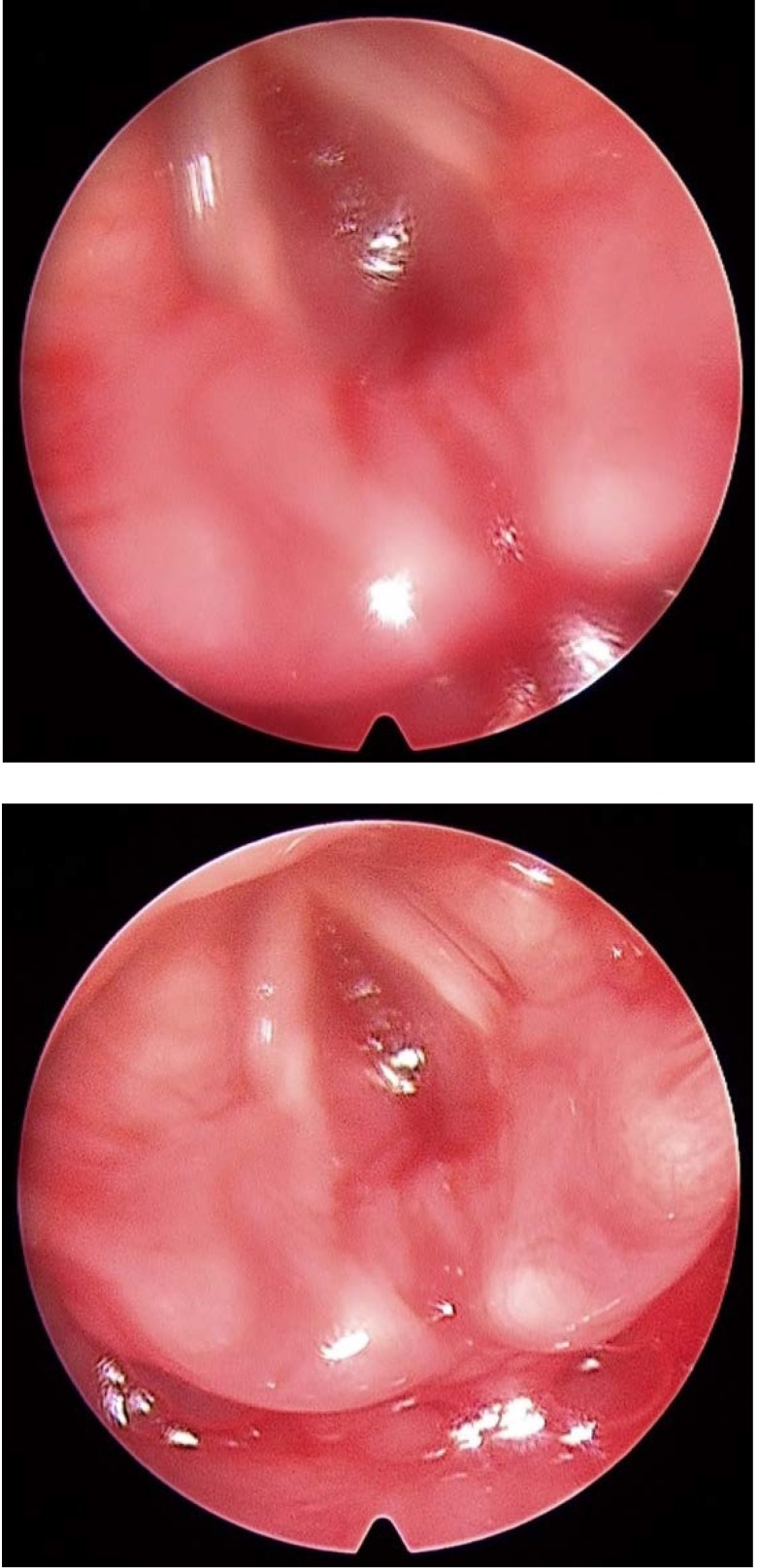
Subglottis completely obstructed, in spite of a large opening of the vocal cords free margins

A direct laryngoscopy revealed a severe (grade III) subglottic stenosis (**Fig. 1,2**). It was considered a result from prolonged intubation needed for the previous surgery. No imaging was consequently performed. A partial cricotracheal resection was decided and surgery was undertaken.

**Fig. 3:**
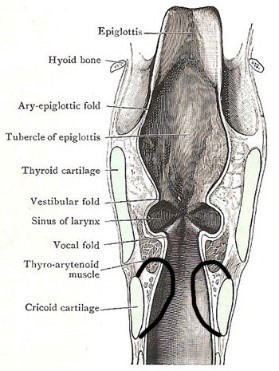
Scheme of the subglottis narrowing discovered intraoperatively

Intraoperatively, no mucosal stricture/ stenosis could be elicited at the subglottic level. Instead, a concentric narrowing of the whole subglottic region was observed (**Fig. 3**).

Partial laryngofissure was performed, up to the level of the anterior commissure. Care was taken to preserve the relative position of the vocal cords to each other. The anterior midline section of the cricoid and region palpation showed an hourglass shaped cartilage ring, with a nearly complete obstruction of the airway. Instead of the resection of the cricoid, we decided to drill the cartilaginous excess, after thoroughly dissecting away and preserving the normal mucosa. Afterwards, it was repositioned over the thinned cartilage and sutured to its sectioned margins. The laryngofissure was then approximated and left open as in anterior cricothyrotomy. The tracheal cannula was left in place postoperatively. A 3-days stenting was performed, and the oral fluid intake was allowed, together with antibiotic prophylaxis.

**Fig. 4:**
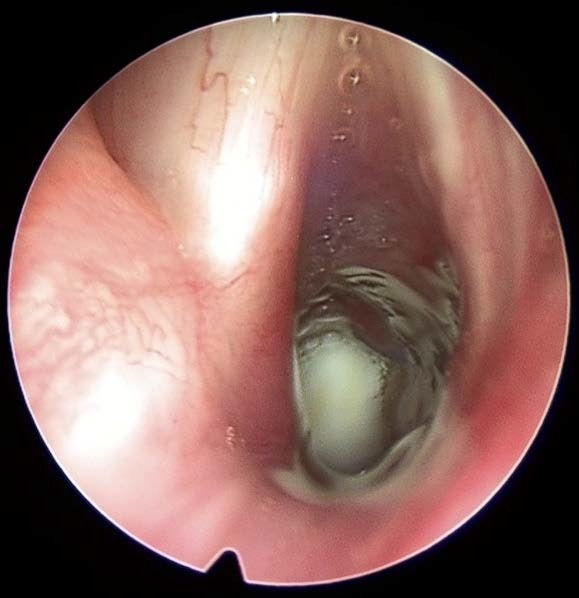
Postoperative endoscopy of the larynx, 13 days after surgery

The airway was subsequently investigated endoscopically 13 days after surgery (**Fig. 4**). It showed a good respiratory laryngeal subglottis, so the patient could be decannulated. 6 months after tracheostomy closure, the patient remained symptom free and came annually for monitoring.

## Discussion

The presented case was a particular one because of his concomitant heart disease. He did not have his laryngeal malformation recognized, in spite of a major surgical procedure performed and investigations undertaken elsewhere. The impossibility of decannulation after a prolonged tracheal intubation usually has iatrogenic significance (acquired subglottic stenosis). There are authors communicating a 24% risk for heart surgery and comatose patients to develop obstructive subglottic lesions, due to long term intubation [**7**].

The severity of our patient stenosis did not allow the preoperative passage/ measuring of a small 2,7mm Hopkins rod at laryngoscopy. That was why we concluded upon the need for surgery.

Many surgical options have been described in literature. Laser and balloon dilation seem the most favored at the moment but cannot be used for a severe disease [**8**]. Anterior cricoid split has longtime been proposed for congenital forms of the disease [**9**]. Cartilage grafting of anterior and/ or posterior cricoid split can be also a choice [**10**]. Still, the best surgical results have been associated with partial cricotracheal resection [**11**]. Although challenging, the technique is well standardized in our department and its use can sometimes represent the best management we can offer to a patient.

Our intraoperative findings of a malformed cricoid cartilage offered 2 options of continuing the surgery: a cricotracheal resection (as planned) or a thorough drilling of the cartilaginous laryngeal frame, with laryngeal mucosa preservation. We performed the latter and used the mucosa to protect the drilled surfaces from infection and a long process of secondary healing. After less than 2 weeks, the maintaining of a good endolaryngeal space allowed the patient decannulation without complications.

The drilling of the laryngeal cartilage has been described before along with cricotracheal resection [**12**]. The concept was found interesting and that was why we decided to apply it in our case. However, the idea can have pros and cons. The preservation of the mucosa can promote the local healing and enhance the chances of success. Still, it cannot be used if it is severely damaged/ scared. It cannot always offer enough material to cover the drilling defect (usually larger than the initial narrow endolaryngeal surface). The drilling of the cricoid cartilage should be carefully performed in order to preserve the integrity of the cricoarytenoid joints. The remnant of the cartilage should also ensure enough support for the muscular pressure that accompanies swallowing. Therefore, it is a fine balance between the surgical gestures, in order to obtain the perfect result.

## Conclusion

Laryngeal malformations represent a wide spectrum of anatomical lesions. Some of them can go unnoticed by the physician, due to lower degrees of severity. Comorbidities can contribute to the clinical emphasis of the laryngeal problem. A detailed imaging of the larynx is always necessary in order to point out the local anatomy and severity of malformations. Laryngeal surgery remains an important tool for a significant number of congenital and acquired subglottic stenosis. The decision to use a certain technique or the other is based on the individual patient and the surgeon’s preferences. Endolaryngeal shaping (submucosal drilling) of the malformed laryngeal cartilages can be of value in selected cases. The external approach and the operating microscope ensure the best exposure conditions.
